# Data fusion-based traffic prediction and software decision support for recreational suburban roads

**DOI:** 10.1371/journal.pone.0343224

**Published:** 2026-05-05

**Authors:** Shahriar Afandizadeh, Saeid Abdolahi, Hamid Mirzahossein

**Affiliations:** 1 School of Civil Engineering, Iran University of Science and Technology, Tehran, Iran; 2 School of Civil Engineering, Iran University of Science and Technology, Tehran, Iran; 3 Department of Civil - Transportation Planning, Imam Khomeini International University, Qazvin, Iran; Kafkas University: Kafkas Universitesi, TÜRKIYE

## Abstract

Predicting traffic flow on mountainous suburban roads is challenging due to highly variable environmental, temporal, and traffic-related conditions. This study focuses on Kandovan Road, a critical route with complex behavioral patterns influenced by weather conditions, calendar events, and road-specific characteristics. To improve forecasting accuracy, eight machine learning and deep learning models were implemented, including Deep LSTM, Random Forest Regressor, XGBRegressor, Transformer, ST-ResNet, Conv-LSTM, Bidirectional LSTM, and LSTM-GAN. The models were trained and evaluated using traffic, weather, and event datasets from 2017 to 2023, with performance measured through MAE, RMSE, MSE, and MAPE metrics. Among the evaluated models, the Random Forest Regressor achieved the highest accuracy with an R² score of 0.88 and a low average error. This result demonstrates its strong ability to model non-linear and dynamic traffic patterns. The results indicate that integrating diverse data sources significantly enhances traffic prediction performance on mountain roads. Additionally, a dedicated traffic forecasting software system was developed to visualize real-time predictions and provide an operational decision-support tool for traffic authorities. The outcomes of this work support more efficient traffic management, improved road safety, and sustainable transportation planning in challenging terrains.

## Introduction

The continued growth of human populations and the increasing movement between urban and suburban areas present considerable challenges to road infrastructure [1]. Recent studies highlight the need for strategies that maintain efficient road network performance. One approach involves intelligent systems that integrate neural networks with communication technologies to optimize resources, improve service quality, and reduce operational costs [2]. With the expansion of existing roads becoming increasingly difficult, the development of effective traffic flow management technologies has become essential. Traffic management strategies are therefore critical for reducing congestion, enhancing transportation efficiency and safety, and addressing environmental concerns [3,4]. Additionally, the dynamic nature of transportation data demands adaptive methods. These methods must be capable of responding to evolving traffic patterns and concept drifts in data distributions [5].

However, most prior studies have focused on urban road networks, while suburban roads have received limited attention. Moreover, many studies analyze influencing factors separately, resulting in an incomplete view of their interactions. This underscores the need for integrated models capable of capturing nonlinear and multidimensional traffic behavior. In this work, learning-based approaches are used to identify complex relationships among weather conditions, calendar events, and road-specific variables for traffic flow forecasting. Weather affects traffic capacity, speed, and network efficiency [6], while calendar events such as public holidays and seasonal patterns influence travel demand and driver behavior [7]. Accordingly, selecting suitable forecasting algorithms depends on the contextual characteristics and available data [8].

**Fig 1:**
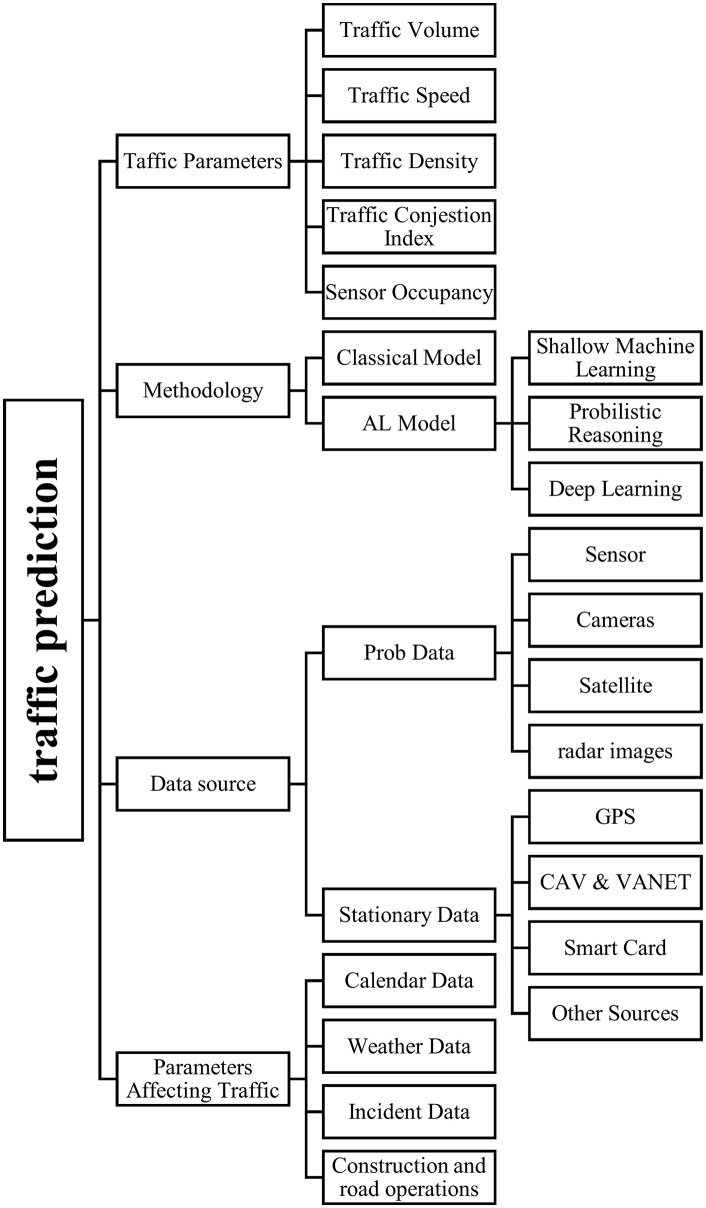
Traffic forecasting system plan.

To address this gap, the present study analyzes traffic flow on Kandovan Road (Fig 1), a suburban mountain route connecting Tehran and Chalus. The road’s narrow structure, hazardous segments, infrastructural limitations, and limited capacity cause severe congestion, especially during holidays. These conditions lead to issues such as time loss, excessive fuel consumption, traffic violations, and unauthorized road usage [9].

By examining previous studies, this article describes the research methodology employed in the case study and presents the findings and implications of the research. The main contributions of this study are as follows:

1. Proposing a hybrid learning framework that integrates heterogeneous data sources to model nonlinear traffic behavior;
2. Comparing the performance of eight state-of-the-art machine and deep learning algorithms for traffic prediction;
3. Developing a native software application that operationalizes the best-performing models and provides an intelligent action plan for real-time traffic management.

By advancing a comprehensive, data-driven approach to traffic prediction, this research contributes to sustainable and intelligent transportation planning in complex suburban environments.

**Fig 2:**
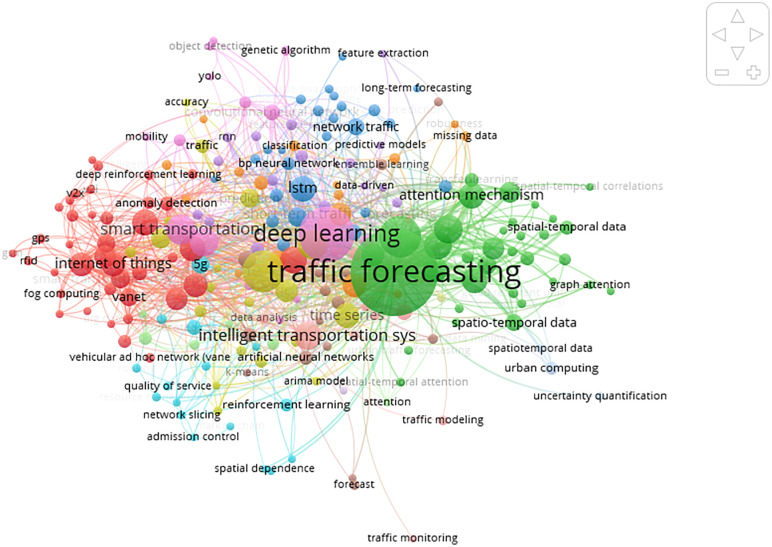
The most prevalent keywords are found in traffic forecasting literature.

Fig 2 illustrates the scientometric analysis of this article and the most common methods used in recent years.

## Literature review

Predictions of short-term traffic patterns are crucial for the effective management and operation of intelligent transportation systems. Nevertheless, predicting traffic flow remains a significant challenge because transportation networks are unpredictable and complex. Over time, a variety of approaches have been developed to tackle this issue, encompassing both classic statistical models and cutting-edge deep learning methods. Classical statistical approaches primarily use time series analysis to predict traffic parameters like density, volume, and speed. In contrast, deep learning methods have become powerful tools for capturing the non-stationary characteristics of traffic data. These techniques can be categorized into parametric and non-parametric approaches, each offering distinct benefits based on the specific conditions and needs [10,11]. Applying model results across different data sets (localization) is challenging due to varying driving behaviors and traffic characteristics across locations [12], rendering the choice of approach highly dependent on local conditions.

Lv et al. (2014) explored the general features of traffic flow using a stacked autoencoder model trained with a layer-wise greedy algorithm [13]. In this deep learning-based prediction model, the inherent relationships between spatial and time-dependent traffic patterns are taken into account. Predicting any traffic characteristic requires both historical data from the area and real-time data from surrounding regions. In 2017, Liu et al. colleagues introduced a novel deep learning framework that integrates convolutional and LSTM modules to capture spatio-temporal patterns in traffic flow [14]. Additionally, Bi-directional LSTM model was employed to analyze previous traffic data in order to identify the recurring patterns of traffic movement.

Also in 2017, Zhang et al. employed a neural network architecture to model the time-based, recurring, and trend-related aspects of traffic, utilizing three convolutional layers to record trend, periodic, and nearby data from citywide traffic images [15]. During the same year, Yu et al. used long short-term memory (LSTM) networks (LSTM) and an autoencoder to capture ordered dependencies, particularly for peak hours and post-crash scenarios, making their approach suitable for traffic prediction under extreme conditions [16]. Although these models account for temporal or spatial dependencies, most fail to capture both simultaneously.

In 2018, Cheng et al. integrated road structure with CNN and RNN models to predict traffic congestion; subsequently, Yao et al. introduced a deep multiview spatio-temporal network architecture to model spatial-temporal relationships from a temporal, spatial, and semantic perspective [17]. This model simultaneously learns spatio-temporal dependencies by combining Long Short-Term Memory, local convolutional neural networks (CNN), and semantic encoding. To address the dynamic nature of traffic, long-term forecasting has employed spatio-temporal convolutional neural networks (STCNNs) with convolutional short-term memory units.

In 2020, Fan et al. employed a model that captures spatial-temporal correlations based on past traffic data to facilitate long-term forecasts [18]. In 2021, Yin et al. explored multi-level attention-driven graph-based spatio-temporal models for traffic prediction, incorporating a self-attention mechanism to capture correlations between various time series from the same data source, along with a dynamic attention framework based on neighborhood data to model spatial interactions [19].

Predicting traffic in rural networks poses a unique challenge due to irregular temporal patterns. In 2022, Hamim et al. applied hybrid methods, utilizing aggregation methods and the ordinal logit (OL) framework, to obtain the most precise predictions across various periods for road segments [20]. In 2023, Chen et al. introduced a GSTPRN (Graph-based Spatio–Temporal Position Recurrent Network) for predicting traffic conditions [21], while Lablack and Shen introduced a novel attention mechanism with Spatio-temporal graph MixFormer to model the relationships between spatial and temporal dependencies [22]. Rasaizadi et al. in 2024 focused on managing traffic big data dimensions to enhance short-term traffic patterns on suburban routes [23]. In 2024, Afandizadeh et al. utilized calendar data to predict traffic patterns on suburban routes [9]. Finally, Harrou et al. in 2024 developed a powerful deep learning model that combines wavelet-based noise reduction with recurrent neural networks (RNNs) for enhanced performance [24]. Table 1 provides an overview of studies related to the topic of the present research.

**Table 1:** An overview of traffic flow prediction articles.

Number	Reference & Year	Dataset	Focus	Result	Evaluation Index	Method
#1	Lv et al. [13]	PeMS	A new deep learning-driven method for predicting traffic flow is presented	takes into consideration the intrinsic spatial and temporal dependencies	MAE = 34.1 RMSE = 50	SAE
#2	Zhang et al. [15]	TaxiBJ & BikeNYC	An ST-ResNet-based system is devised to harness the essential spatial and temporal dynamics in predictive modeling	Utilizes a data-driven approach to combine outputs from multiple residual neural networks, dynamically adjusting weights for specific branches and regions	RMSE = 6.33	ST-ResNet
#3	Yu et al. [16]	real-world	Leverage Deep LSTM to anticipate peak-hour congestion and distinguish key attributes of traffic data.	Present an innovative approach for model interpretation through signal activation	MAPE = 0.97	Mixture Deep LSTM
#4	Cheng et al. [25]	real-world	Present a complete framework named DeepTransport, which integrates Convolutional Neural Networks (CNN) and Recurrent Neural Networks (RNN) to analyze spatio-temporal traffic patterns within a transportation network configuration.	Illustrate that this technique effectively learns intricate spatial and temporal interactions.	RMSE = 0.45	CNN -RNN
#5	Yao et al. [17]	Didi Chuxing	Introduce a framework called Deep Multi-View Spatial-Temporal Network (DMVST-Net) designed to capture both spatial and temporal relationships	Combines the spatio-temporal, and semantic perspectives, modeled using local CNN, LSTM, and semantic graph embeddings.	MAPE = 0.16 RMSE = 9.64	CNN -LSTM
#6	Yin et al. [19]	PeMS	Create a spatial attention model to represent changing spatial relationships over time.	A dynamic attention mechanism is employed to identify spatial dependencies, both within individual neighborhoods and between distinct neighborhoods.	RMSE = 24.23 MAE = 15.62	MASTGN
#7	Hamim et al. [20]	video data	Developing a model based on empirical data to assess and predict the capacity of highway roundabouts, considering both geometric design and traffic conditions.	Developing a model based on empirical data to assess and predict the capacity of highway roundabouts, considering both geometric design and traffic conditions.		multivariate regression analysis
#8	Chen et al. [21]	real-world	Develop a position-based graph convolution module using self-attention to capture the spatial dependencies between nodes	Integrate graph convolution based on position, personalized propagation approximation, and adaptive learning of graphs into a recurrent network mode	RMSE = 24.96 MAE = 15.68	GSTPRN
#9	Lablack and Shen, [22]	real-world	1. Creating an adaptive adjacency matrix through attention-based and similarity-driven learning. 2. Variable fields of view applied in temporal convolution to achieve a smaller number of parameters	Both the estimation and the distance matrix are provided as input to the attention mechanism	RMSE = 2.62 MAE = 1.25	STGM
#10	Rasaizadi et al. [23]	Maintenance and Transportation Organization’s website	Forecast the traffic conditions on a specific segment of a suburban roadway	Using cyclic patterns in traffic prediction models results in greater accuracy than models that only utilize the original set of features.		Svm, Lstm, Rf
#11	Afandizadeh et al. [9]	Rmto Iran	Analyze calendar-based data to sustain the dynamic nature of traffic movement and enhance the accuracy of current traffic prediction models.	The fusion of machine learning techniques with calendar insights and roadway characteristics facilitates precise forecasting of traffic patterns, considering daily and situational variations in congestion level	MAE = 86.72 RMSE = 148.35	Random forest regressor
#12	Harrou et al. [24]	Traffic measurements from diverse California highway locations	For better prediction performance, traffic flow data is preprocessed through exponential smoothing and wavelet filtering, ensuring the removal of anomalies	introduces a deep learning framework that enhances traffic flow prediction by combining wavelet-based denoising with RNN architectures, including LSTM and GRU	R^2^ = 0.982	LSTM and GRU

Previous studies have primarily employed machine learning and deep learning models such as LSTM, CNN, ST-ResNet, and hybrid architectures for traffic flow prediction. These works have aimed to capture spatiotemporal traffic dependencies and enhance prediction accuracy. Some studies, such as those by Zhang and Cheng, have specifically focused on combining convolutional and recurrent networks to model spatiotemporal patterns. Moreover, a significant portion of prior research has relied on datasets like PeMS, video-based traffic data, or urban transportation systems, which are mostly collected from large, structured urban road networks. Thus, the main commonality between these studies lies in their reliance on advanced learning models to extract traffic patterns and their efforts to improve prediction accuracy. However, most previous studies have paid insufficient attention to the specific road and environmental conditions of mountainous and intercity areas, and often incorporated only one or two data types (e.g., traffic or weather data) into their models. Furthermore, in many studies, the simultaneous effects of calendar events, seasonal conditions, weather variables, and traffic behaviors on highly volatile road segments have been overlooked, or the models have been evaluated primarily on relatively stable urban networks.

## Materials and methods

A review of the literature highlights a research gap in the application of deep learning and machine learning models for traffic flow prediction, particularly on suburban roads as opposed to conventional urban networks. However, previous studies have generally paid insufficient attention to the specific road and environmental conditions of mountainous and intercity areas, often incorporating only one or two data types (e.g., traffic or weather data) into their models. Furthermore, many studies either failed to consider the simultaneous effects of calendar events, seasonal conditions, weather variables, and traffic behaviors on highly volatile road segments, or the models were implemented on relatively stable, structured urban networks. The present research addresses this issue by simultaneously combining traffic data, weather information, calendar events, and road features for the specific and highly volatile Kandovan Road. Additionally, the research findings have been implemented as a native Decision Support System (DSS) software, which allows for practical use in real-time traffic management an aspect that was neglected in prior studies. This study also seeks to tackle the challenges arising from the diversity and dispersion of the case study data. From a statistical standpoint, the research integrates datasets from road and transport authorities to enhance the reliability of findings.

Given the characteristics of the study area (discussed in Section 4), the modeling process involves developing a dynamic prediction model that synthesizes various aspects of the problem, following data extraction and feature engineering. The model is subsequently fine-tuned to match the specific characteristics of the case study dataset.

After reviewing relevant studies and analyzing the case study characteristics, this research employs several machine learning algorithms, including LSTM, Transformer, XGBoost Regressor, St-ressNet, and Random Forest Regressor. LSTM is chosen for its effectiveness in modeling time-series data, XGBoost and Random Forest for their robust performance with structured datasets and high interpretability, Transformer for its ability to capture both long-term and short-term dependencies, and StressNet for its specialized design in handling complex and multi-dimensional data. The combination of these algorithms aims to enhance prediction accuracy and uncover intricate relationships among variables.

**Fig 3:**
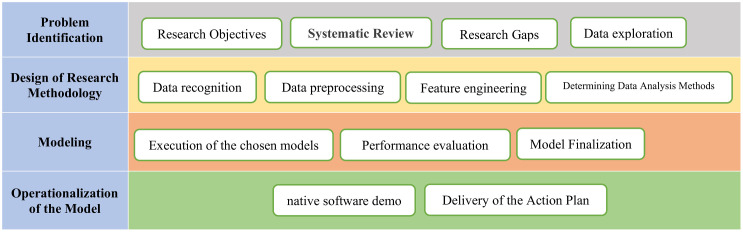
Research performance stages.

To optimize the performance of the selected algorithms, systematic hyperparameter tuning methods such as Grid Search and Random Search are applied. Each algorithm’s hyperparameters are adjusted individually: for LSTM, the number of layers and neurons; for Transformer, the number of attention heads and embedding dimensions; for XGBoost, the number of trees and tree depth; for StressNet, the number of convolutional layers and filters; and for Random Forest, the number of trees and tree depth. The final model is constructed using data gathered from multiple sources over a historical period. Preprocessing steps ensure the data is well-structured and suitable for analysis. The parameters, variables, and constraints of the model are determined based on the collected dataset and aligned with insights from the systematic literature review (SLR) on traffic prediction methodologies. The most appropriate solution algorithm is then selected to address the research problem effectively. Once the model is built and trained with the collected data, validation is carried out by comparing its predictions with real-world observations. Standard validation techniques are applied, including analysis of current conditions, identification of potential discrepancies, and evaluation of model performance. Sensitivity analysis is also conducted to evaluate the robustness of the predictions. Finally, a predictive model is developed for forecasting future traffic conditions, with its accuracy assessed using standard evaluation metrics such as Mean Squared Error (MSE). Fig 3 illustrates the overall modeling framework.

In this article, three algorithms -LSTM, RandomForest Regressor, and XGBRegressor- based on the data characteristics used, have been chosen based on the characteristics of the available data. These algorithms are evaluated and discussed in further detail in subsequent sections.

### LSTM

Long Short-Term Memory (LSTM) networks are a specialized type of Recurrent Neural Network (RNN) designed to address the limitations of traditional RNNs in handling long-term dependencies [26–28]. At the heart of an LSTM network is the memory cell, which manages information flow through a system of gates. These gates, composed of sigmoid layers and multiplication operations, regulate the addition or removal of information. A gate output of zero blocks information transfer, while an output of one allows complete transfer. LSTMs utilize three primary gates to control the memory cell’s state.

The operation of an LSTM network begins with the forget gate, which generates a value between zero and one based on the input X_t_ and the previous hidden state $h_{\left(t-\text{1}\right)}$. This value determines how much of the previous cell state C_(t-1)_ is retained for the current state C_t_ [29]. Mathematically, the forget gate f_t_ is expressed as:


$$f_{t}=\sigma \;\left(W_{t}.\left[C_{t-\text{1}},h_{t-\text{1}},x_{t}\right]+b_{t}\right)$$ (1)

Next, the input gate decides what new information will be stored in the memory cell. This involves applying both a sigmoid function and a hyperbolic tangent (tanh) function to the input data. The results are combined with the output of the forget gate to update the cell state. The updated cell state C_t_ is computed as:


$$C_{t}=f_{t}*C_{t-\text{1}}+i_{t}*\tilde{C_{t}}$$ (2)

Here, it represents the input gate, and $\tilde{C_{t}}$ is the candidate cell state, calculated as:


$$i_{t}=\sigma \;(W_{i}.\left[h_{t-\text{1}},x_{t}\right]+b_{i}$$ (3)

$$\tilde{C_{t}}=tanh\;(W_{c}.\left[h_{t-\text{1}},x_{t}\right])+b_{c})\;$$ (4)

Finally, the output gate determines which part of the cell state will be passed to the output. This is achieved by applying a sigmoid function to the input and hidden state, followed by a tanh operation on the updated cell state. The output gate $o_{t}$and the final hidden state $h_{t}$ are computed as:


$$o_{t}=\sigma \;(W_{o}.\left[C_{t-\text{1}},h_{t-\text{1}},x_{t}\right]+b_{o}$$ (5)

$$h_{t}=o_{t}*\;tanh\;(C_{t})$$ (6)

Through this gating mechanism, LSTM networks effectively manage information flow, enabling them to learn and retain long-term dependencies in sequential data.

### RandomForest regressor

Random Forest is an ensemble learning method that leverages the strengths of Classification and Regression Trees (CART) and the bagging technique (bootstrap aggregation) to overcome common limitations in predictive modeling [30]. The bagging approach involves creating multiple independent CART decision trees and averaging their predictions for regression tasks. This method reduces prediction variance and minimizes bias. In contrast, boosting, another ensemble technique, generates predictions by computing a weighted average of outputs from all trees, with weights adjusted sequentially during training [31]. Unlike boosting, bagging operates in parallel, making it highly efficient for large datasets by leveraging modern multi-core computing systems. For this reason, this study focuses on the bagging method within the Random Forest framework. The Random Forest algorithm introduces randomness in two key aspects to enhance accuracy [32]. First, each CART tree is trained on a bootstrap sample T_b_ (b = 1, ⋯, B) randomly selected with replacement from the full training set T. Samples not included in the bootstrap sample are referred to as “out-of-bag” samples. Second, during the construction of each CART tree, a random subset of M features is selected from the total P available features (where M < *P*). The optimal split for each tree is determined using the randomly chosen T_b_ and M features. The ensemble of trees can be represented as:


$$\left\{\phi T_{b,m}|b=\text{1},\ldots ,B\right\}$$ (7)

For regression tasks, the final prediction is obtained by averaging the outputs of all trees:


$$\;\;\hat{\;\;\;\;\;Y=}\;\phi_{T,P}(X)=\frac{\text{1}}{B}\;(\sum_{b=\text{1}}^{B}\phi T_{b,m}(X))$$ (8)

When the output variable y_n_ is a single-value variable, the above equations suffice to build a Random Forest regressor. However, in cases where the output variable consists of multiple values, such as:


$$y_{n}=\;\{y_{n,\text{1}},\;\ldots ,\;\;y_{n,q},\;\ldots ,\;y_{n,Q}\}$$ (9)

the Random Forest technique can handle data correlation through two approaches. The first approach involves a Multiple Output Regression Chain (MORC), which extends the traditional single-output Random Forest method [33]. The MORC method constructs a regression chain by reintroducing the last predictor y_q_ into the original feature set x, creating an augmented feature set:


$$x_{n}\cup \;y_{n,\text{1}:q}=\;\left(x_{n\text{1}},\;x_{n\text{2}},\;\ldots ,\;x_{nP\;},\;\ldots ,y_{n,\text{1}}\;,\;\ldots ,y_{n,q}\;\right)$$ (10)

In this setup, the predictor becomes:


$$y_{n}=\{yn,q+\text{1}\}\;$$ (11)

The regression chain progresses sequentially, starting from y_n,1_ and advancing step-by-step until y_n,Q_.This approach accounts for data correlation, as y_n,q+1_ depends on the preceding predictors y_n,1_,…,y_n,q_*.*

### XGBoost regressor

This study employs Extreme Gradient Boosting (XGBoost), an advanced and optimized variant of the Gradient Boosting Method (GBM) [34]. While traditional Decision Trees treat all features as a single system, ensemble methods like Bagging improve decision-making by combining multiple decision trees that “vote” to produce more accurate predictions. Random Forests further enhance this approach by integrating Bagging with Decision Trees and using a random subset of features to increase robustness. Building on these foundations, the Gradient Boosting Method (GBM) refines tree-based algorithms through an iterative gradient optimization process. XGBoost takes this a step further by incorporating software and hardware optimizations, significantly improving performance, speed, and flexibility for both regression and classification tasks. The mathematical formulation of XGBoost is expressed as:


$$\overset{\left(t\right)}{\underset{i}{\hat{y}}}=\;\sum_{k=\text{1}}^{t}f_{k}\left(x_{i}\right)={\hat{y}}^{\left(t-\text{1}\right)}i+\;f_{t}\left(x_{i}\right)\;$$ (12)

Where:

- y^i(t)*:* is the predicted crash severity after the t_*t*_ iteration, obtained by adding a new tree function to the prediction from the (t − 1)th iteration for the i-th crash.
- K: represents the number of additive trees.
- t: is the number of iterations.
- f_k_(xi): is the k-th tree function applied to the input features xi*.*
- y^i^(t−1)^: is the predicted value from the previous iteration.
- f_t_(xi): is the tree function added in the t-th iteration.

### Transformer

The Transformer architecture marks a significant breakthrough in deep learning, particularly for tasks like machine translation and natural language processing (NLP). Unlike traditional models that rely on recurrent or convolutional layers, Transformers are built entirely on attention mechanisms. The key components of a Transformer are self-attention and multi-head attention. Self-attention allows each element in a sequence to interact with all other elements, capturing dependencies regardless of their distance. Multi-head attention extends this by running multiple self-attention mechanisms in parallel, enabling the model to learn diverse and complex relationships within the data [34]. This design has proven highly effective in modeling intricate patterns and dependencies, making Transformers a powerful tool for a wide range of applications [35].

### St-resnet

The Spatio-Temporal Residual Network (ST-ResNet) was originally designed to predict citywide crowd flows, enabling authorities to anticipate future movement patterns across different urban areas. This capability is crucial for assessing public safety and implementing timely preventive measures. Crowd flow data exhibits unique spatio-temporal characteristics: spatially, the inflow into a specific area is influenced by outflows from both nearby and distant regions; temporally, it is affected by recent intervals and demonstrates gradual changes over extended periods [36].

The ST-ResNet architecture is structured around three primary components, each addressing distinct temporal aspects: closeness, periodicity, and trends. Traffic flow data, such as average speed and volume, is first transformed into a matrix resembling a single-channel image. In this matrix, the x-axis represents various traffic variables, while the y-axis corresponds to specific road segments. The time axis is segmented to reflect data from recent, near-history, and distant-history intervals.

- Closeness: Data from the most recent hours is used to model short-term changes.
- Periodicity: Information from the previous day and two days prior captures recurring patterns.
- Trends: Data from more distant time periods helps identify long-term trends.

The aggregated data is normalized to the range [−1,1] using the Tanh function, which accelerates convergence during backpropagation. The three components share a common network structure, consisting of a convolutional neural network (CNN) followed by Residual Units. These units are particularly effective at capturing spatial dependencies across different regions, making ST-ResNet a powerful tool for analyzing and predicting spatio-temporal data.

### Bidirectional LSTM

A bidirectional LSTM analyzes the input sequence in both chronological and reverse order, allowing the model to understand temporal relationships in both directions. A study by Greff et al. (2016) demonstrated that bidirectional LSTMs enhance the performance of speech recognition systems. The output of a bidirectional LSTM is formed by merging the results of two independent LSTMs one processing the sequence forward and the other backward. This combined output encapsulates information from both preceding and subsequent contexts. The outputs of the two LSTMs are merged at each time step to generate the final result. The Bi-LSTM formulas are as follows [37]:

First, forward hidden states are calculated using:


$$\overset{f}{\underset{t}{h}}=f(w_{f}*\;\left[\overset{f}{\underset{t-\text{1}}{h}}-x_{t}\right]+b_{f}$$ (13)

$$\overset{f}{\underset{t}{c}}=\overset{f}{\underset{t}{i}}*g\left(\overset{f}{\underset{c}{w}}*\left[\overset{f}{\underset{t-\text{1}}{h}}.x_{t}\right]+\overset{f}{\underset{c}{b}}\right)+\overset{t}{\underset{f}{f}}*\;\overset{f}{\underset{t-\text{1}}{c}}$$ (14)

$$\overset{f}{\underset{t}{o}}=\;\sigma \;(\overset{f}{\underset{o}{w}}*\;\left[\overset{f}{\underset{t-\text{1}}{h}}.x_{t}\right]+\;\overset{f}{\underset{o}{b}}$$ (15)

$$\overset{f}{\underset{t}{y}}=\;\overset{f}{\underset{t}{o}}*\;\overset{f}{\underset{t}{h}}$$ (16)

Where:

- $\overset{f}{\underset{t}{h}}$: Forward hidden state at time step *t*
- $\overset{f}{\underset{t}{c}}$: Forward cell state at time step *t*
- $\overset{f}{\underset{t}{i}},\overset{t}{\underset{f}{f}},\overset{f}{\underset{t}{o}}$: Input, forget, and output gates, respectively
- *g*: Activation function for the cell state
- $w_{f},\overset{f}{\underset{c}{w}},\overset{f}{\underset{o}{w}},b_{f},\overset{f}{\underset{c}{b}},\overset{f}{\underset{o}{b}}\;$: Learnable parameters

Next, backward hidden states are calculated similarly:


$$\overset{b}{\underset{t}{h}}=f(w_{b}*\;\left[\overset{b}{\underset{t-\text{1}}{h}}-x_{t}\right]+b_{f}$$ (17)

$$\overset{b}{\underset{t}{c}}=\overset{b}{\underset{t}{i}}*g\left(\overset{b}{\underset{c}{w}}*\left[\overset{b}{\underset{t-\text{1}}{h}}.x_{t}\right]+\overset{b}{\underset{c}{b}}\right)+\overset{b}{\underset{f}{f}}*\;\overset{b}{\underset{t-\text{1}}{c}}$$ (18)

$$\overset{b}{\underset{t}{o}}=\;\sigma \;(\overset{b}{\underset{o}{w}}*\;\left[\overset{b}{\underset{t-\text{1}}{h}}.x_{t}\right]+\;\overset{b}{\underset{o}{b}}$$ (19)

$$\overset{b}{\underset{t}{y}}=\;\overset{b}{\underset{t}{o}}*\;\overset{b}{\underset{t}{h}}$$ (20)

Where:

- $\overset{b}{\underset{t}{h}}$: Backward hidden state at time step *t*
- $\overset{b}{\underset{t}{c}}$: Backward cell state at time step *t*
- $\overset{b}{\underset{t}{i}},\overset{b}{\underset{f}{f}},\overset{b}{\underset{t}{o}}$: Input, forget, and output gates, respectively
- $w_{f},\overset{b}{\underset{c}{w}},\overset{b}{\underset{o}{w}},b_{f},\overset{b}{\underset{c}{b}},\overset{b}{\underset{o}{b}}\;$: Learnable parameters

Finally, the forward and backward hidden states are concatenated to produce the final output for each time step:


$$y_{t}=\left[\overset{f}{\underset{t}{y}},\;\overset{b}{\underset{t}{y}}\right]$$ (21)

This allows the network to incorporate information from both past and future contexts as it processes each element in the input sequence.

### Conv-LSTM

Integrating convolutional layers with LSTM layers enables the model to discern local patterns within sequences and enhances feature extraction. Yao et al.‘s 2020 [38] study highlighted that merging CNNs with LSTMs boosts time series forecasting accuracy. CNNs utilize convolutional layers to derive features from input data through filters, generating feature maps. When paired with LSTM layers, CNNs serve as feature extractors for sequential data, allowing the CNN-LSTM model to identify intricate Spatio-temporal patterns by amalgamating spatial and temporal data. This architecture is particularly beneficial for sequential data with spatial/temporal structures, aiming to extract significant features.

In the Conv-LSTM model, an input sequence of length “T” is assumed, with each element represented by *x*_*t*,_ and a filter bank of size *k* with learnable parameters [26,39]. The convolution operation’s output at time step *t* is:


$$Y_{t}=Conv(x_{t},\;W)$$ (22)

Where:

- Y_t_: Output of the convolution operation at time step *t*.
- W: Learnable filter weights.

Then, using the following equations, the input, forgetting and output gates are calculated:


$$i_{t}=\;\sigma \;(Conv\left(Y_{t},U_{i}\right)+\;Conv\left(h_{t-\text{1}},V_{i}\right)+b_{i})$$ (23)

$$f_{t}=\;\sigma \;(Conv\left(Y_{t},U_{f}\right)+\;Conv\left(h_{t-\text{1}},V_{f}\right)+b_{f})$$ (24)

$$o_{t}=\;\sigma \;(Conv\left(Y_{t},U_{o}\right)+\;Conv\left(h_{t-\text{1}},V_{o}\right)+b_{o})$$ (25)

Then, the state of the candidate cell is calculated using the convolution operation:


$$g_{t}=\;tanh\;(Conv\left(Y_{t},U_{g}\right)+\;Conv\left(h_{t-\text{1}},V_{g}\right)+b_{g})$$ (26)

Finally, the cell state and the hidden state are updated using input, forget, and output gates:


$$c_{t}=\;f_{t}*c_{t-\text{1}}+\;i_{t}*g_{t}$$ (27)

$$h_{t}=\;o_{t}*\text{tanh}(c_{t})$$ (28)

Where:

- $i_{t},f_{t},o_{t}\;$: Input, forget, and output gates, respectively.
- U,V,b: Learnable parameters for gates and candidate states.
- $c_{t}$: Cell state at time step *t*.
- $h_{t}$: Hidden state at time step *t*.

This architecture enables the model to extract Spatio-temporal features, making it suitable for tasks like video prediction and time-series forecasting.

### LSTM-GAN

This section describes the proposed LSTM-GAN method for predicting. It employs a trajectory prediction approach, where historical features are used as input to predict the future trajectory of the feature. The LSTM-GAN predictor model uses an LSTM layer to extract features and a fully connected layer to predict the feature value at the next time step. To address the challenge of limited training data, a Generative Adversarial Network (GAN) is used to generate synthetic data [40]. The GAN consists of:

- Generator: Generates synthetic data with a similar probability distribution to the real data.
- Discriminator: Distinguishes between real data (xi) and generated synthetic data (G(zi)), where zi is random noise.

The objective function of the discriminator is:


$$\text{Max}\left(V_{D}\right)=\;\frac{\text{1}}{n}\;\sum_{i=\text{1}}^{n}[\text{log}(D\left(x_{i}\right)+\text{log}\text{1}-\;D\left(G\left(z_{i}\right))\right)]\;$$ (29)

where D(xi) is the discriminator’s output for real data xi.

The objective function of the generator is:


$$\text{Min}\left(V_{G}\right)=\;\frac{\text{1}}{n}\;\sum_{i=\text{1}}^{n}\text{log}(\text{1}-\;D\left(G\left(z_{i}\right)\right)$$ (30)

The training of the GAN involves iteratively optimizing VD and VG. The LSTM GAN predictor model and the GAN are jointly trained to achieve more robust predictions.

### Performance evaluation

To assess the effectiveness of various traffic forecasting methods, four key evaluation metrics are implemented: Root Mean Square Error (RMSE), Mean Absolute Percentage Error (MAPE), Mean Absolute Error (MAE), and Mean Squared Error (MSE). These metrics are defined as follows [41]:

A. **Root Mean Square Error (RMSE)**

RMSE quantifies the average deviation of the model’s predictions from the actual values. A lower RMSE indicates better predictive accuracy.


$$\text{RMSE}=\sqrt{\frac{\text{1}}{\text{n}}\sum_{\text{i}=\text{1}}^{\text{n}}{\left({\text{y}}_{\text{i}}-\overset{―}{{\text{y}}_{\text{i}}}\right)}^{\text{2}}}$$ (31)

B. **Mean Squared Error (MSE)**

MSE represents the average squared difference between predicted and actual values, providing insight into the magnitude of prediction errors.


$$\text{MSE}=\frac{\text{1}}{\text{n}}\sum_{\text{i}=\text{1}}^{\text{n}}{\left({\text{y}}_{\text{i}}-\overset{―}{{\text{y}}_{\text{i}}}\right)}^{\text{2}}$$ (32)

C. **Mean Absolute Error (MAE)**

MAE measures the average absolute difference between predicted and actual values, reflecting the model’s overall error magnitude.


$$\text{MAE}=\frac{\text{1}}{\text{n}}\sum_{\text{i}=\text{1}}^{\text{n}}\left|{\text{y}}_{\text{i}}-\overset{―}{{\text{y}}_{\text{i}}}\right|$$ (33)

D. **Mean Absolute Percentage Error (MAPE)**

MAPE expresses the error as a percentage of the actual values, making it useful for understanding relative accuracy.


$$\text{MAPE}=\frac{\text{1}}{\text{n}}\sum_{\text{i}=\text{1}}^{\text{n}}\frac{\left|{\text{y}}_{\text{i}}-\overset{―}{{\text{y}}_{\text{i}}}\right|}{\left|\text{y}\right|}*\text{1}\text{0}\text{0}$$ (34)

In these equations:

$y_{i}$: represents the ground truth (actual value).

$\overset{―}{y_{i}}$: denotes the predicted value.

n: is the number of observed samples.

## Case study

Kandovan Road, officially known as Road 59, serves as a crucial route connecting Tehran and Karaj to northern Iran, extending from Karaj, located in Alborz province, to Chalus, a city in Mazandaran. This 180-kilometer mountain road links Tehran to the Caspian Sea coast, winding through stunning yet rugged mountain landscapes, making it a major tourist attraction. Kandovan Road is characterized by steep inclines, tight turns, narrow lanes, and was originally built in the 1930s, with multiple renovations undertaken to enhance safety and accessibility. The road’s technical features include numerous tunnels and bridges that navigate the rugged terrain. Despite these challenges, it continues to serve as a crucial route in northern Iran. The road experiences a variety of weather conditions, including clear, cloudy, foggy, rainy, snowy, and stormy weather, and due to its winding nature and avalanche risks, accidents are frequent.

**Fig 4:**
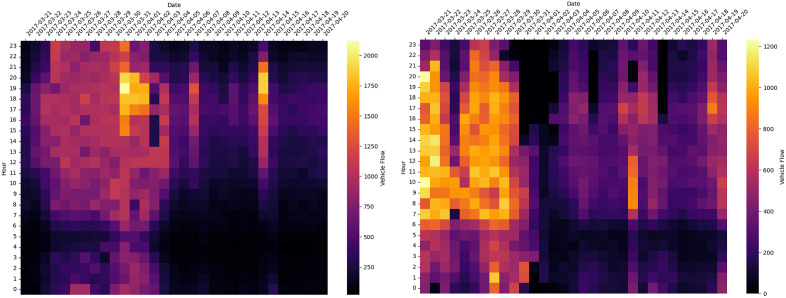
Chalus-Tehran, Tehran- Chalus: Heatmap representation of traffic flow over the analyzed time frame.

**Fig 5:**
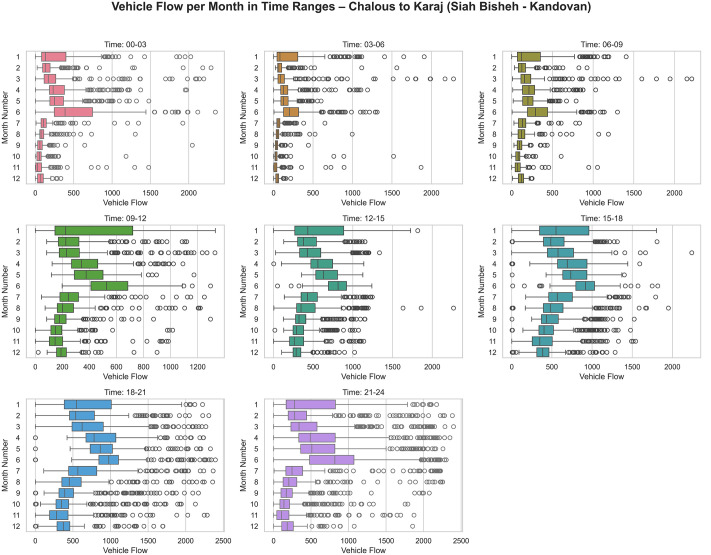
Box plot illustrating traffic flow along the Chalus-Tehran route over the analyzed time frame.

The study focuses on the section between Kandavan Tunnel and Siah Bisheh, with traffic flow data displayed through heatmap and boxplot (Figs 4 and 5) for different periods. The data shows significant flow dispersion during high-traffic periods, such as Nowruz holidays and summer peaks. Peak traffic volume occurs during the evening to nighttime hours (especially between 18–21 and 21–24), during which the median and spread of traffic flow are significantly higher compared to other time periods. The early months of the Solar Hijri year exhibit higher traffic levels across most time intervals compared to other months, likely attributable to increased road trips during the Nowruz holidays.

Conversely, the late night to early morning hours (00–06) experience the lowest traffic volumes, corresponding with natural sleep and travel patterns. The data distribution in peak traffic intervals (such as 15–24) shows high variability, revealing substantial fluctuations in traffic flow during these periods.

Additionally, heat maps illustrate traffic dynamics during Persian New Year, with peak flows from Tehran to Chalus, while the reverse direction remains blocked. Likewise, in the three days leading up to the end of the holiday, traffic from Chalus to Tehran becomes heavier, while the Tehran-bound lane remains closed.

**Fig 6:**
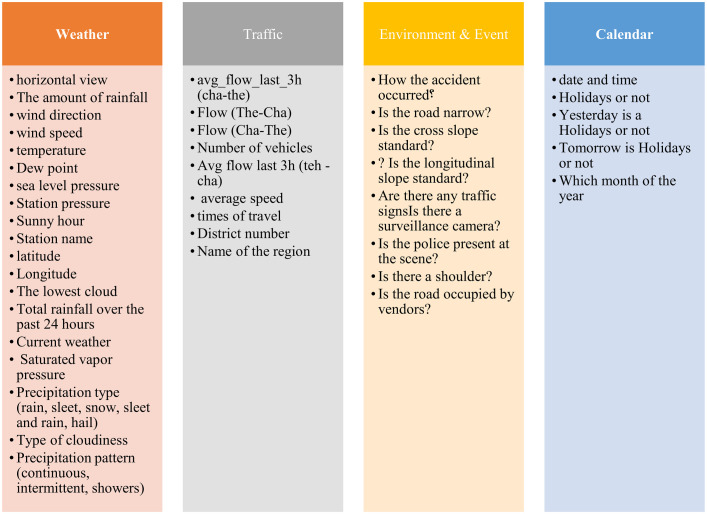
Variables used in the research.

The traffic flow data used in this study were obtained from the official traffic counting system of the Ministry of Roads and Urban Development of Iran. This dataset includes continuous hourly traffic counts recorded by automatic sensors installed along the Kandovan suburban corridor between 2017 and 2023. Weather data were collected from the Meteorological Organization and the corresponding Siah Bisheh Road synoptic station, and holiday/event information was extracted from the Iranian national calendar. The data objects are classified into four categories, and the variables used in this study fall within these categories (Fig 6).

## Discussion

### Data preprocessing, hyperparameter tuning

The raw dataset comprises traffic information, weather data, calendar events, and environmental event data. The following preprocessing steps were applied to prepare the data for analysis:

Missing values were addressed using methods such as interpolation and replacement with the mean. In cases where the number of missing values was minimal, corresponding rows or columns were deleted. Additionally, missing values were imputed using the average of preceding and succeeding data points. To address data imbalance, resampling techniques were applied to ensure a more balanced distribution of the dataset. Textual data, such as region names, were converted into categorical numerical representations through One-Hot Encoding to make them suitable for machine learning algorithms. Numerical features, including wind speed, temperature, and traffic volume, were normalized to a standard range. This step was essential to enhance the performance and convergence of the algorithms. Noisy data were either removed or corrected using methods such as Moving Average filters and statistical techniques, including the elimination of data points outside the valid range. Time-based data were transformed into 3-hour intervals. Specifically, traffic data initially recorded at 1-hour intervals were aggregated to align with this new format. Correlation analysis was employed to identify and remove features that were highly correlated with each other. Additionally, non-useful or low-impact features were eliminated to streamline the dataset and improve model efficiency. Following preprocessing and feature extraction, principal component analysis (PCA) was applied to reduce the dataset’s dimensionality.

The hyperparameters listed in Table 2 were selected through a systematic tuning process. Grid Search was applied to LSTM-based and Transformer models to evaluate different combinations of layer depth, neuron counts, batch size, and learning rate. Random Search was used for Random Forest and LSTM-GAN due to their larger parameter spaces. Model configurations were evaluated using a validation split, and the final values were chosen based on the lowest validation MAE and RMSE, ensuring model stability and preventing overfitting.

**Table 2:** A Comprehensive Overview of Hyperparameter Optimization Across Models.

Model	Optimization Technique	Key Hyperparameters		value
LSTM	Grid Search	Configuration Details	Epochs	100
			Batch size	32
			Neurons	64
			Layers	3
Random Forest	Random Search		Min samples split	2
			Max depth	15
			Trees	200
XGBoost	Grid Search		Trees	10
			Learning rate	0.05
			Trees	300
Transformer	Grid Search		Embedding dimension	64
			Attention heads	8
ST-ResNet	Manual tuning		Filters	64
			Residual units	4
Conv-LSTM	Grid Search		LSTM units	64
			Kernel size	3 × 3
			Conv filters	32
Bi-LSTM	Grid Search		Units	128
			Layers	2 (bidirectional)
LSTM-GAN	Random Search		Batch size	64
			Epochs	200

by utilizing learning approaches, the study aimed to predict traffic flow based on factors such as weather, calendar schedules, and notable events. To measure the accuracy of the model, the target parameter was analyzed using error metrics including MSE, RMSE, MAE, and MAPE. MSE measures squared prediction errors, RMSE provides error values in their original measurement units, and MAE calculates the average absolute error, MAPE is an essential indicator for assessing the accuracy of predictive models. These metrics facilitate the comparison of different models, helping to identify the most accurate one.

### Analysis results

Training, testing, and validation were conducted on the selected models (Deep LSTM, RandomForest Regressor, XGBRegressor, Transformer, ST-ResNet), with 80% of the data allocated for training and 20% for testing. Python 3 on Jupyter Notebook 6.0.3 was used for computations, with data spanning from 2017 to 2023. The objective was to forecast hourly traffic flow over a 24-hour period. Results show the RandomForest Regressor outperformed other models, achieving an RMSE of 139.21 for the Chalus-Tehran route and 135.38 for Tehran-Chalus, suggesting moderate predictive accuracy. The MSE values for these routes were 16815.16 and 18326.55, respectively, reflecting variability in predictions, while MAE values of 67.19 (Chalus-Tehran) and 77.71 (Tehran-Chalus) suggested relatively low average prediction errors. The Random Forest Regressor algorithm, with a MAPE value of 0.24, has the lowest mean absolute percentage error among all algorithms. This indicates that the average deviation between the predicted and actual values is only 0.24%.

The Transformer model exhibited the weakest performance, with error values of 214.92 and 148.43 for the respective routes. The RandomForest Regressor achieved R² values of 0.83 and 0.78 for the test set and validation, respectively, indicating a better fit and closer approximation to actual values.

This superiority can be justified from several perspectives. First, the data used in this study are non-stationary, highly volatile, and strongly influenced by environmental conditions. Sudden changes caused by holidays, mountainous weather, accidents, and road capacity limitations lead to unstable temporal patterns, rendering many intervals unsuitable for deep learning models. Deep learning models usually require large volumes of homogeneous and consistent data to achieve optimal performance. However, mountain road traffic data especially during peak periods and special days exhibit high noise, abrupt fluctuations, and limited repeating patterns.

Comparing the results of this study with previous works (e.g., Lv et al., 2014; Yu et al., 2017; Yao et al., 2018) indicates that deep learning models tend to perform better in urban traffic networks where patterns are more stable and abundant data are available. In contrast, in contexts where the road network is limited and travel behavior is strongly affected by national calendars and unpredictable weather conditions (such as in Effendizadeh et al., 2024), tree-based models generally provide more stable and accurate performance.

**Fig 7:**
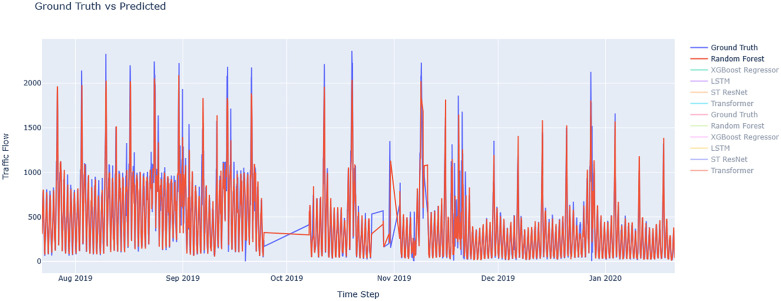
Evaluation of Chalus-Tehran Traffic Flow with the Random Forest Regressor Model.

**Fig 8:**
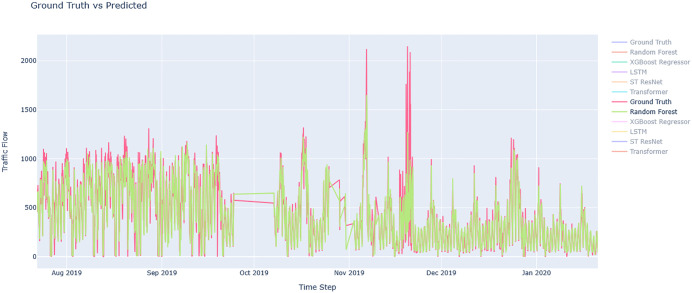
Evaluation of Tehran – Chalus. Traffic Flow with the Random Forest Regressor Model.

**Fig 9:**
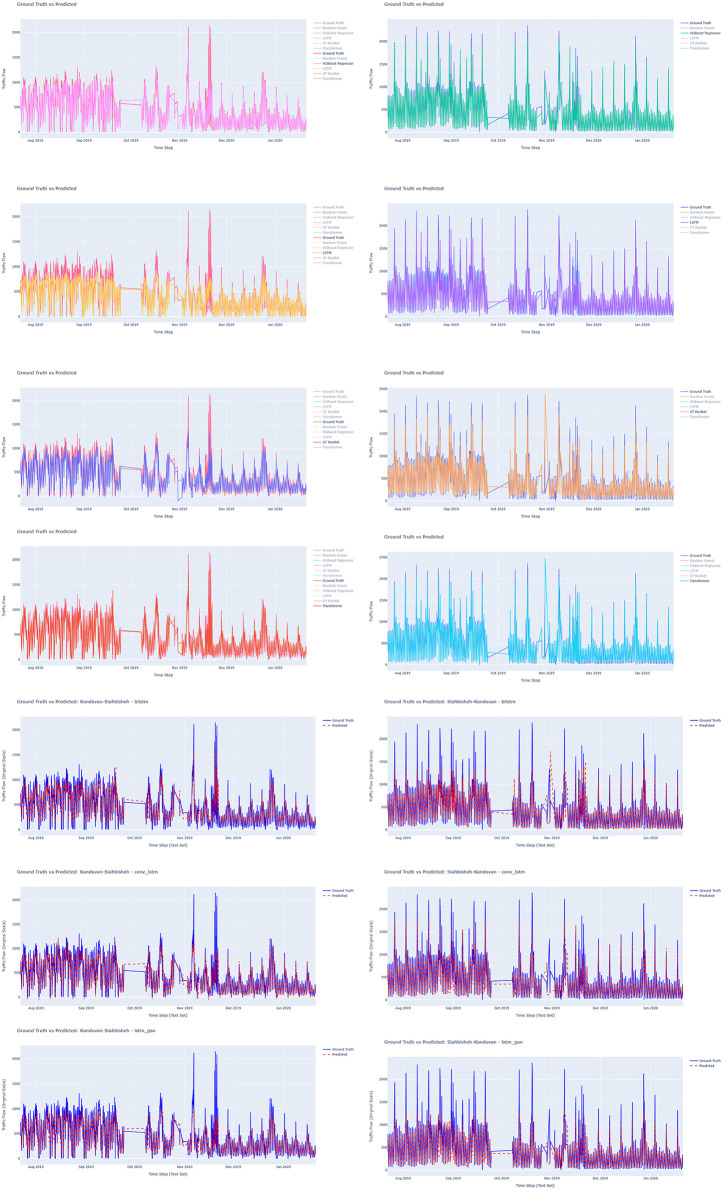
Comparing the actual and predicted value of traffic flow using all models on the route.

Figs 7 and 8 depict the prediction accuracy for round-trip routes for RandomForest Regressor model that had a superior fit to actual observations. Fig 9 compares the actual and predicted traffic flow values using all the available models along the study route.

**Fig 10:**
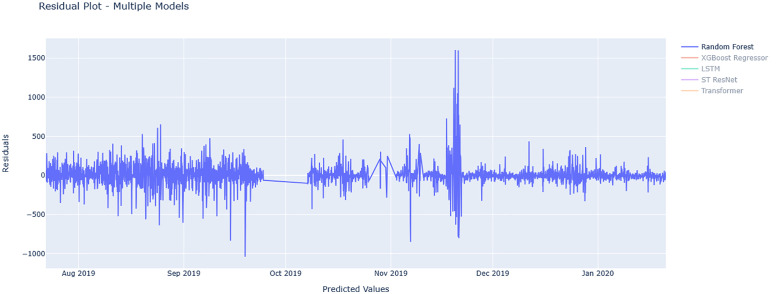
Residual value for Tehran-chalus route for random forest model.

**Fig 11:**
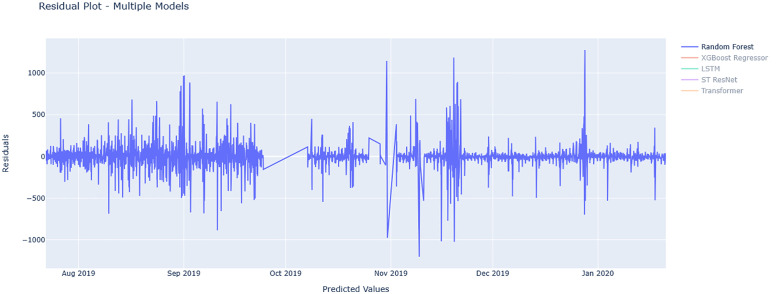
Residual value for Chalus-Tehran route for random forest model.

**Fig 12:**
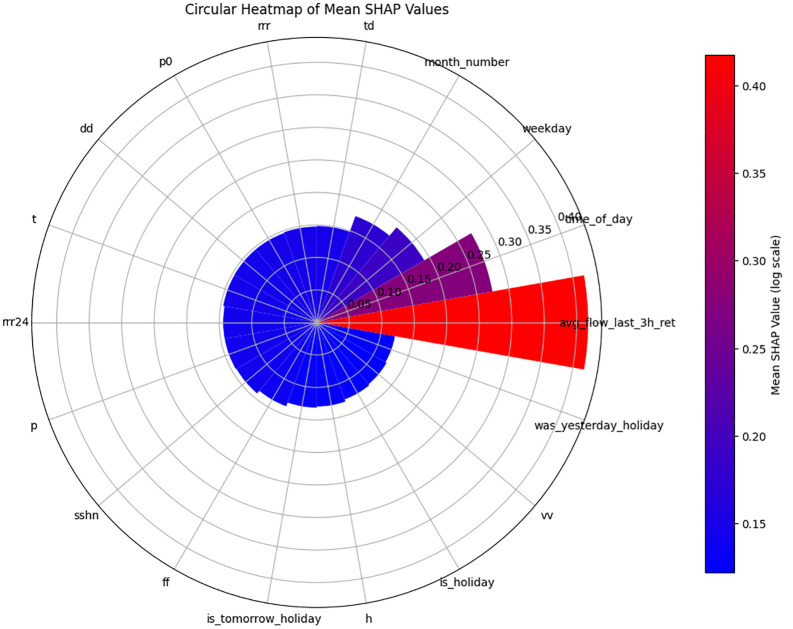
Investigating variables affecting traffic flow in the Random Forest model for Tehran to Chalus route.

**Fig 13:**
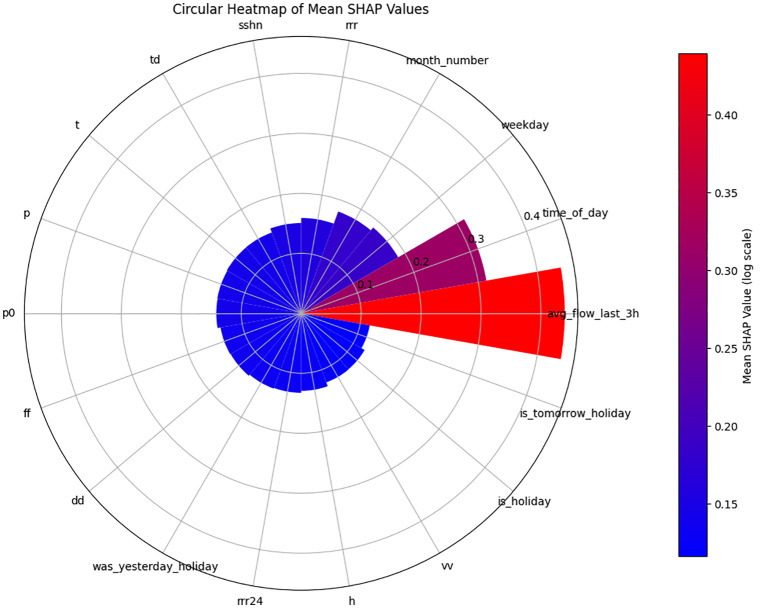
Investigating variables affecting traffic flow in the Random Forest model for Chalus to Tehran route.

**Fig 14:**
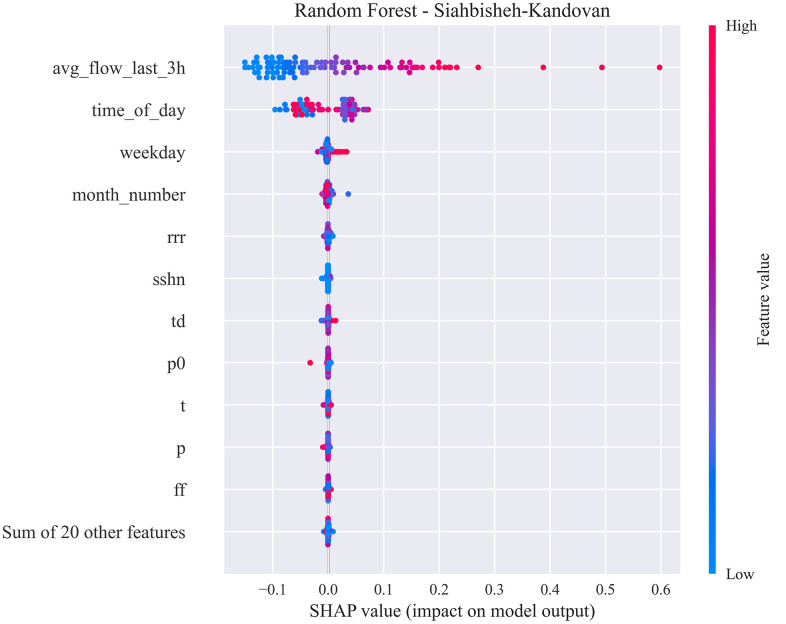
Shap value traffic flow in the Random Forest model for Chalus to Tehran route.

**Fig 15:**
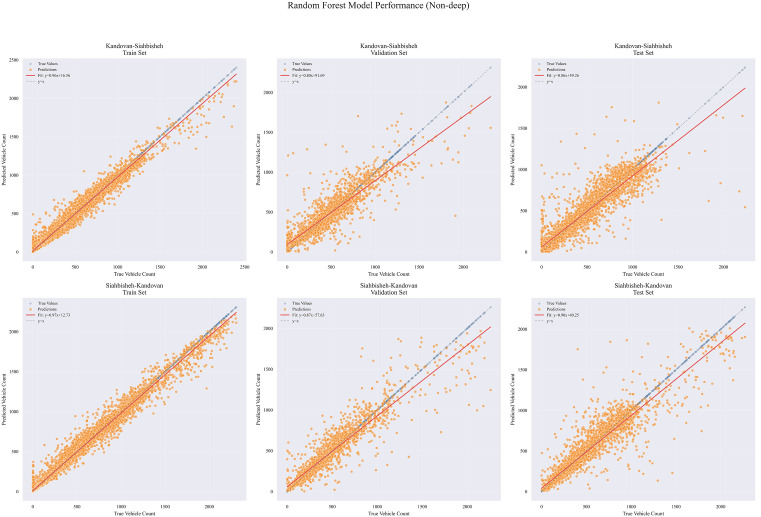
Investigating variables affecting traffic flow in the Random Forest model for Chalus to Tehran route.

Figs 10 and 11 show residual value differences between actual and predicted values, while Figs 12 and 13 depict the impact of various factors on traffic flow in a Random Forest model. Given that Random Forest is not inherently a time-series model, three-hour traffic data was included as a variable to enhance prediction. Fig 14 illustrates the effects of influential variables after conducting modeling operations by Shap value. These figures indicate that the time of day after the traffic flow in the last three hours variable is the most critical factor in predicting traffic flow on Kandovan Road in a RF model. This indicates that recent traffic conditions and time of day are key factors in determining the intensity of traffic flow on the route under consideration. Additionally, other static variables that lack an influence factor and are not displayed in Figs 12 and 13 were eliminated during the modeling process. Fig 15 illustrates the performance of the Random Forest model in estimating traffic flow for the two high-traffic routes: Kandovan–Siahbisheh and Siahbisheh–Kandovan, across three data sets: training, validation, and test. The model demonstrates satisfactory performance across both routes and all three data subsets, as indicated by the coefficient of determination values, which reflect a strong correlation between the predicted and actual values. Even in the test set, despite a relatively higher dispersion of points, the model maintains an acceptable level of accuracy, indicating good generalization capability. Overall, the results of this analysis highlight the robustness of the Random Forest model in capturing nonlinear and complex traffic data patterns, especially under highly volatile and non-stationary conditions. These findings can assist decision-makers in more effective planning, especially considering events’ impact on traffic patterns. The results confirm the model’s efficacy in predicting traffic flow, although further refinements could improve prediction accuracy.

**Fig 16:**
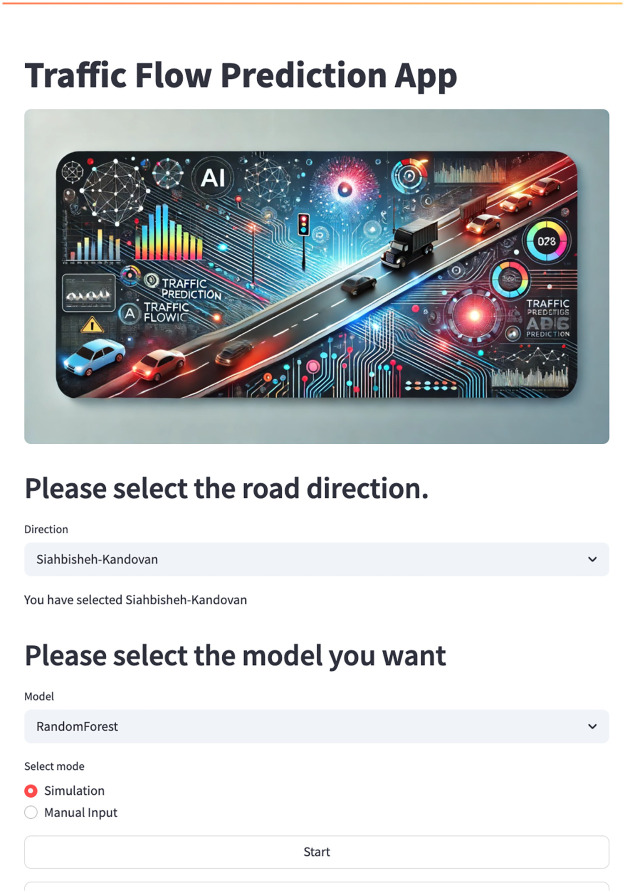
Traffic native software demo.

The study also developed a system to visualize predictions and actual data using a native traffic forecasting software (https://github.com/saeid1993/trefficflow). Fig 16 shows the User Interface (UI) of this software. The UI has three modules. The first module displays the route. The second module determines which model is desired. And the third module specifies whether the modeling is based on previous data or whether we provide new data to the desired parameters ourselves. Table 3 shows the Model performance evaluation indicators for Test data. Table 4 summarizes the test-set performance metrics of the models, grouped into Top, Mid-Performing, and weakest models.

**Table 3:** Model performance evaluation indicators for Test data.

Model	Direction	Dataset	R2	MAE	RMSE	MAPE (%)	MSE	AIC	BIC
Random Forest	Kandovan-Siahbisheh	Train	0.98	23.73	40.57	46.23	1645.57	198812.7	199064.6
		Validation	0.78	93.47	151.45	133.56	22936.85	27829.78	28011.41
		Test	0.83	77.71	135.38	104.50	18326.55	48212.40	48412.21
	Siahbisheh-Kandovan	Train	0.99	20.86	41.38	24.73	1711.99	199580.4	199832.3
		Validation	0.86	77.66	139.21	46.26	19380.67	27466.56	27648.19
		Test	0.88	67.19	129.67	24.55	16815.16	47884.91	48084.72
XGBoost	Kandovan-Siahbisheh	Train	0.96	44.01	65.30	94.77	4264.10	217286.1	217538
		Validation	0.78	93.69	153.17	134.14	23461.1	27878.50	28060.14
		Test	0.83	78.12	134.80	108.81	18170.33	48179.83	48379.64
	Siahbisheh-Kandovan	Train	0.97	35.84	57.65	48.81	3323.67	212451.9	212703.9
		Validation	0.84	81.27	151.95	49.80	23090.23	27844.15	28025.78
		Test	0.87	69.01	135.55	24.031	18376.04	48222.66	48422.47
LSTM	Kandovan-Siahbisheh	Train	0.86	72.24	115.53	223.92	13347.30	314893.1	612554.7
		Validation	0.78	97.64	153.99	146.59	23712.85	103365.1	317846.4
		Test	0.83	82.16	135.18	126.38	18274.08	123666.9	359679.4
	Siahbisheh-Kandovan	Train	0.84	94.77	141.35	175.21	19979.66	322717.0	620378.6
		Validation	0.82	109.50	159.61	62.13	25475.8	103519.2	318000.5
		Test	0.86	104.33	161.25	52.30	26001.04	125006.3	361018.8
StressNet	Kandovan-Siahbisheh	Train	0.86	78.11	119.64	249.87	14314.68	3948794	18545549
		Validation	0.77	103.31	155.91	167.11	24306.70	3735962	14253715
		Test	0.81	88.47	141.38	146.21	19989.24	3756551	15330151
	Siahbisheh-Kandovan	Train	0.85	91.56	140.78	134.14	19818.14	3955103	18551858
		Validation	0.82	112.72	162.01	66.49	26244.47	3736127	14253880
		Test	0.81	102.87	165.38	45.83	27346.35	3757741	15331342
Transformer	Kandovan-Siahbisheh	Train	0.83	83.43	129.51	259.47	16772.74	309691.7	569438
		Validation	0.70	112.67	177.17	158.17	31389.13	94335.81	281497.1
		Test	0.79	92.81	148.43	131.08	22030.44	114744.9	320694.8
	Siahbisheh-Kandovan	Train	0.69	131.82	199.85	251.06	39938.56	326518.5	586264.9
		Validation	0.64	153.43	228.15	94.17	52051.21	95422.70	282584
		Test	0.67	141.22	214.92	84.36	46189.59	117556.7	323506.6
Bi-LSTM	Kandovan-Siahbisheh	Train	0.84	88.08	126.78	319.91	16073.67	426786	1150711
		Validation	0.72	118.75	171.37	218.03	29368.03	212112.8	733739.9
		Test	0.80	98.58	145.48	207.01	21163.65	232512.5	806504.3
	Siahbisheh-Kandovan	Train	0.85	77.01	137.69	98.97	18957.31	429986.3	1153911
		Validation	0.78	105.58	175.41	60.36	30770.26	212213	733840.1
		Test	0.81	90.63	163.93	35.69	26874.30	233419.8	807411.5
Conv- LSTM	Kandovan-Siahbisheh	Train	0.85	78.80	121.50	184.82	14762.38	464047.5	1341145
		Validation	0.72	113.54	171.10	139.81	29276.17	251018.0	883014.3
		Test	0.81	92.11	143.51	123.80	20595.85	271321.2	966761.8
	Siahbisheh-Kandovan	Train	0.85	84.94	136.73	158.18	18693.35	468626.4	1345723
		Validation	0.76	112.18	185.85	66.91	34539.36	251373.3	883369.6
		Test	0.83	94.5854	155.5626	41.6947	24199.737	271933.64	967374.23
LSTM -GAN	Kandovan-Siahbisheh	Train	0.82	83.94	133.37	218.61	17788.36	319919.9	615440.1
		Validation	0.67	121.25	186.45	166.86	34763.28	103643.2	316581.5
		Test	0.79	94.72	149.73	146.15	22418.65	123899.3	358213.9
	Siahbisheh-Kandovan	Train	0.75	92.94	177.74	98.22	31592.24	331059.8	626579.9
		Validation	0.60	144.83	240.22	68.59	57708.21	104732.4	317670.7
		Test	0.72	107.7	198.16	33.37	39267.14	126028	360342.6

**Table 4:** Model performance evaluation indicators for Test data.

Rank	Level	Model	Direction	R² (Test)	RMSE (Test)	Confidence Interval 95% (RMSE)		Key Notes
						(low)	(up)	
1	Top models	Random Forest	Kandovan-Siahbisheh	0.88	129.67	127.87	131.47	Best balance of accuracy and stability
2		XGBoost	Kandovan-Siahbisheh	0.87	135.55	133.67	137.43	Similar to RF, faster execution
3	Mid-Performing models	StressNet	Siahbisheh-Kandovan	0.86	161.25	159.07	163.43	Good for complex patterns
4		LSTM	Kandovan-Siahbisheh	0.83	135.18	133.30	137.06	Requires more tuning
5		Conv LSTM	Kandovan-Siahbisheh	0.8282	155.56	153.37	157.75	Higher error than top models
6		BLSTM	Siahbisheh-Kandovan	0.8093	163.93	161.68	166.18	Average performance
7	The weakest model	LSTM GAN	Siahbisheh-Kandovan	0.7213	198.16	195.46	200.86	Lowest R² among deep models
8		Transformer	Siahbisheh-Kandovan	0.6722	214.92	211.99	217.85	Worst performance in all metrics

## Action plan

**Fig 17:**
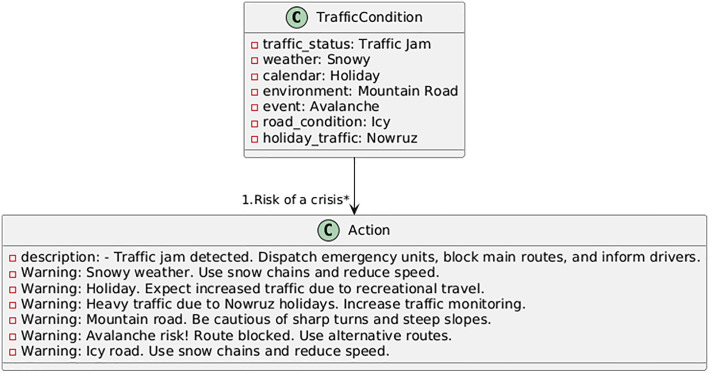
Action plan under traffic congestion conditions.

**Fig 18:**
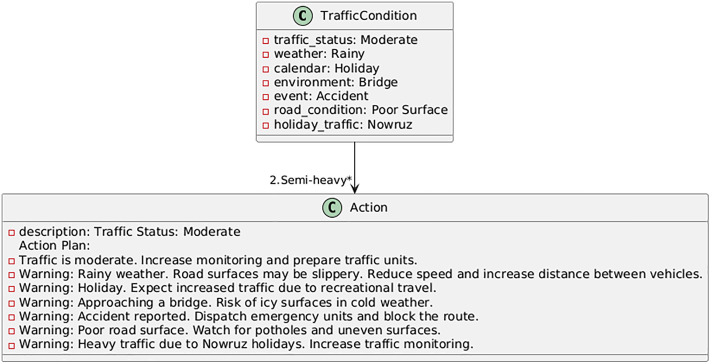
Action plan under moderate traffic conditions.

**Fig 19:**
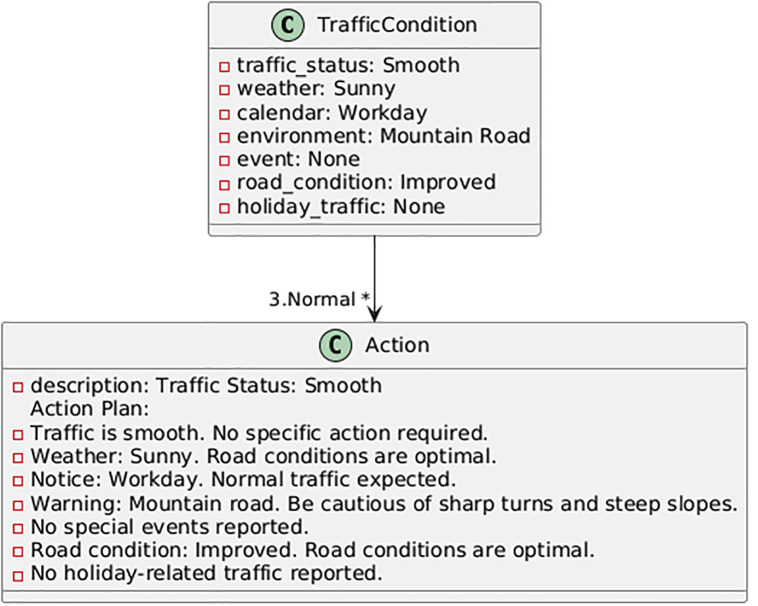
Action plan under normal traffic conditions.

**Fig 20:**
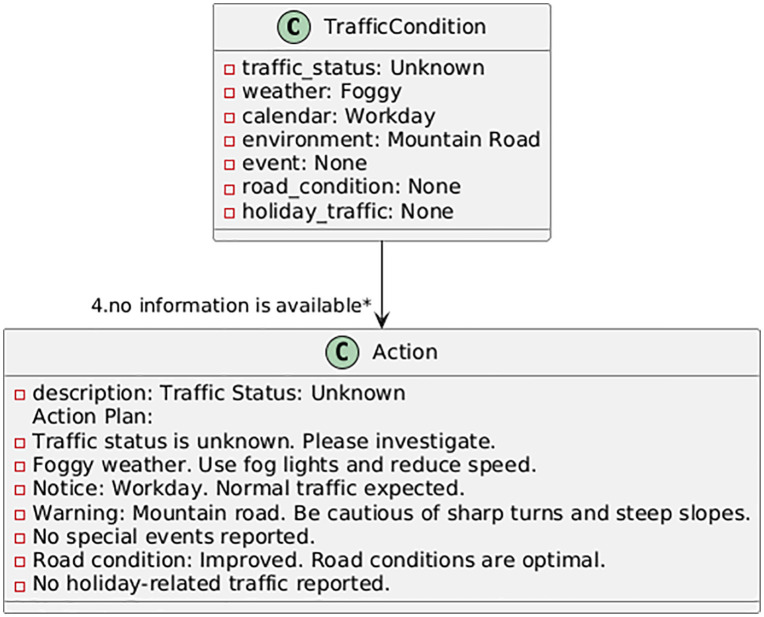
Action plan under uncertain traffic conditions.

In this section, based on the developed models and software interface, an action plan for the designated mountain route is proposed using machine learning language. The action plan outlines steps and operational strategies aimed at improving the safety, efficiency, and sustainability of mountain roads. In addition to addressing current challenges, it anticipates future issues and provides sustainable, intelligent solutions for mountain road traffic management. The proposed plan begins with continuous monitoring of traffic, weather, and event information from multiple sensors and public databases; it advances to the decision-making stage via real-time forecasting using the best-performing models (Random Forest and XGBoost); at this stage, automated decision thresholds trigger control actions such as lane management, dynamic speed limits, or traveler information messages; and finally, the system utilizes response and feedback to evaluate the outcome of interventions and adjust its parameters dynamically. Therefore, the goal of this effort is to address existing problems and anticipate future challenges. By leveraging modern technologies and data analysis tools, this plan enables intelligent traffic management on roads. Using the PlantUML program, some of the action plans are displayed in Figs 17–20. The PlantUML code represents class diagram.

## Conclusion

This study delves into the intricate challenge of predicting traffic flow along Kandovan Road, a vital artery linking Tehran to the Caspian Sea region. Known for its limited capacity and persistent congestion, especially in Tehran-bound traffic, accurate forecasting is critical for effective road management and long-term planning. Moving beyond traditional approaches that focus narrowly on traffic metrics, this research introduces a holistic traffic flow performance index to evaluate the overall health of the road network. To improve prediction precision, the study integrates a wide array of data sources, including traffic logs, weather patterns, and major event records, utilizing state-of-the-art machine learning and deep learning techniques. Recognizing that mountainous, two-lane roads like Kandovan differ markedly from modern highways, being more vulnerable to weather disruptions and seasonal shifts, this research seeks to refine forecasting methodologies by incorporating these critical factors. Traffic forecasts for both the Tehran-Chalus and Chalus-Tehran directions were generated using advanced models such as Long Short-Term Memory (LSTM), Random Forest Regressor, XGBoost Regressor, Transformer, Spatio-Temporal Residual Networks (ST-ResNet), convolutional-LSTM, Bidirectional LSTM and LSTM-GAN. These models were rigorously trained, tested, and validated using performance metrics like Mean Absolute Error (MAE), Mean Squared Error (MSE), Root Mean Squared Error (RMSE), and Mean Absolute Percentage Error (MAPE). The findings demonstrate that deep learning and machine learning models excel in capturing short-term variations and long-term patterns, demonstrating robust predictive performance. The Random Forest model demonstrated high accuracy and generalization capability in estimating traffic flow across both directions, proving its effectiveness in modeling complex, nonlinear, and non-stationary traffic patterns.

Moreover, the study addresses the complexities of handling large-scale traffic datasets by employing dimensionality reduction techniques to mitigate overfitting. The results underscore the reliability of the proposed models in delivering accurate and consistent forecasts. Furthermore, this study, through the detailed analysis of data and the behavioral patterns of drivers and traffic, is capable of simulation and prediction various road and traffic conditions, which helps transport authorities, traffic management organizations, and road police make timely decisions based on accurate information. Beyond traffic management, this research plays a key role in enhancing road safety. By improving interactions between road infrastructure, vehicles, and road users, particularly through the utilization of advanced technologies such as intelligent traffic management systems, roadway crises can be mitigated. A custom traffic forecasting software was also developed, embedding the trained models and case study data to serve as a practical decision-support tool. In conclusion, the study presents an intelligent traffic management framework specifically designed for the unique demands of mountainous road networks, providing actionable solutions for more efficient traffic control and improved travel planning.

## Supporting information

S1 File PLOSOne-data. (XLSX)

## List of Abbreviations:

MSE: Mean Square Error
RMSE: Root Mean Square Error
MAE: Mean Absolute Error
MAPE: Mean absolute percentage error.
